# High-Dose Neuromodulators: A Roundtable on Making Sense of the Data in Real-World Clinical Practice

**DOI:** 10.1093/asjof/ojab036

**Published:** 2021-09-19

**Authors:** Sabrina Guillen Fabi, Jean Carruthers, John Joseph, Sue Ellen Cox, Steve Yoelin, Julius Few, Joely Kaufman-Janette, Steven Dayan

**Affiliations:** Department of Ophthalmology, University of British Columbia, Vancouver, BC, Canada; Clinical Testing of Beverly Hills, Encino, CA, USA; University of North Carolina, Chapel Hill, NC, USA; University of Chicago Pritzker School of Medicine, Chicago, IL, USA; Division of Facial Plastic and Reconstructive Surgery, Department of Otolaryngology, University of Illinois at Chicago, Chicago, IL, USA

## Abstract

**Background:**

For aesthetic treatment with botulinum neurotoxin type A (BoNTA), interest in maximizing treatment duration and efficacy has prompted study of doses higher than those used in registration studies. As data emerge, it is important that physicians understand how to apply study findings to their own practice so that patient demand is satisfied.

**Objectives:**

To bring together leading experts in neuromodulators for a roundtable discussion on the implications of high-dose BoNTA studies for patient care.

**Methods:**

The authors reviewed and discussed recent data from high-dose BoNTA studies for abobotulinum toxin A, incobotulinum toxin A, and Oonobotulinumtoxin A.

**Results:**

Discussion focused on the challenges of data interpretation and extrapolation of study findings for real-world patient care. The authors participated in a candid discussion of whether the observed improvements in treatment duration and patient satisfaction warrant treatment with high-dose regimens delivered as high-concentration injections. Safety was also discussed, as well as economic considerations for both practices and patients. Of note, for BoNTA products, the registration dose, when administered in a smaller total volume, appears to give rise to more durable results than those observed in pivotal trials, implicating product concentration as an important consideration. Importantly, at higher doses, extended duration of effect does not appear to be at the expense of natural-looking results.

**Conclusions:**

While the authors provide considerations for the development of individual clinical practice, there is no one-size-fits-all recommendation. It may be that “high-dose” BoNTA is in reality the optimal dose; however, important economic considerations may prevent rapid uptake for all patients.

Clinical preparations of botulinum neurotoxin type A (BoNTA) produce local and functional denervation in injected muscle by inhibiting the presynaptic release of acetylcholine, thereby reducing muscle contraction.^1^ In aesthetics, the capacity of BoNTA to reduce the activity of facial muscles in a controlled and targeted way underpins its utility in managing the appearance of dynamic lines as well as potentially slowing or reducing their development in patients treated earlier in life.^2,3^ Efficacy and duration of response to BoNTAs are dose-dependent, and the dose administered can be adjusted based on volume of diluent used to reconstitute the lyophilized product and/or the total volume of a given dilution injected.^4^ There are 4 currently approved BoNTA products available in the United States, and in the pivotal trials for these agents, the duration of effect for the FDA-labeled dose in glabellar lines is between 3 and 4 months.^5-9^ The clinical profiles of these agents, such as incobotulinumtoxinA (INCO; Xeomin, Bocouture; Merz Pharmaceuticals GmbH, Frankfurt am Main, Germany), onabotulinumtoxinA (ONA; Botox/Vistabel, Allergan Inc., Dublin, Ireland), abobotulinumtoxinA (ABO; Dysport/Azzalure, Ipsen Pharma, Wrexham, UK), and prabotulinumtoxinA (PRA; Nabota, Daewong Therapeutics, Korea/Jeuveau, Evolus Inc., USA/Nuceiva, Evolus Inc., Canada, Europe), are considered to be clinically similar, with limited peer-reviewed evidence of differentiation regarding safety, time to onset, efficacy, or durability.^10^

Though the field of aesthetics has long been interested in tailoring treatment to individual patient needs as well as optimizing treatment outcomes and patient satisfaction by maximizing duration of treatment effect, there has been a recent resurgence in interest surrounding doses that are higher than those approved by the US FDA. Beyond a broader interest in improving patient outcomes, industry interest in conducting so-called “high-dose” studies may, in part, be driven by the 24-week median time to return to baseline (moderate or severe glabellar lines) observed for 40U of daxibotulinumtoxin A (DAX, Revance Therapeutics, Inc, Newark, CA, USA)^11^ in clinical studies, a duration longer than that reported for other available products based on pivotal trial study dosing.^5-9,12^

Dovetailing with the emergence of new products is the recognition that as treatments become more durable, interest from the public increases. This pattern has held true for fillers, and initial anxiety on the part of physicians that longer duration of outcome would disadvantage practices has evolved into an understanding that patients who are satisfied for longer often seek additional treatment. Finally, the exploration of higher BoNTA doses provides a practical advantage, in that a detailed understanding of dose on aesthetic outcomes allows physicians to tailor treatments to individual patient aesthetic goals in an evidence-based fashion.

Today, with high-dose toxin data rapidly emerging, the most pressing questions for practicing clinicians are (1) whether increasing BoNTA dose gives rise to a natural-looking result throughout the treatment course; (2) whether the durability of the result is proportional to the additional product needed; (3) whether increased durability is meaningful to patients; (4) the nature of the economic adjustments required of clinical practices that adopt high-dose regimens; (5) whether the impact of increasing dose is product specific; and (6) whether there is an increased risk of adverse events (AEs) (eg, ptosis). Here, the authors discuss the issues and data impacting the answers to these questions so that clinicians can form an educated opinion on the use of “high-dose” toxin in their own clinical practice.

## METHODS

Together, the authors participated in a 2-hour roundtable discussion on April 10, 2021, during which several aspects of BoNTA dosing were discussed. Each of the authors has extensive experience participating as investigators in multiple BoNTA clinical trials, including those aimed at understanding the impact of increased dose on response, as well as experience in treating a wide range of patients in clinical practice across several geographic areas in the United States and internationally.

## RESULTS

Here, the authors review the most recent data on high-dose INCO, ONA, and ABO and participate in a discussion highlighting considerations for interpretation of study results as well as how they have applied these data to their own practices.^13-16^ This manuscript was prepared under the direction of the authors following the event. Some of the data presented here for ABO and ONA have been presented at medical conferences; however, manuscripts detailing the full findings of these studies are in preparation or submitted to peer-reviewed medical journals at the time of this writing. In keeping with good practices in medical publishing, the data shown here are somewhat limited so that the integrity and the novelty of the published manuscripts are maintained. All patients discussed in this manuscript were treated either as part of a study for which institutional review board approval was obtained or within the author’s practice in accordance with Good Clinical Practice. 

## DISCUSSION

In order to apply findings from clinical studies to practice, one must first interpret the presented data. In the case of BoNTA, this is complicated by variations between products and the clinical studies measuring their efficacy. These challenges are outlined below.

### Interpretation of Evidence From Clinical Studies

The application of clinical study results to real-world clinical practice is an ongoing challenge for practicing physicians. The controlled environment characteristic of a clinical trial demands that a sufficiently narrow patient population is treated and that the treatment (injection pattern, product dose, and product concentration) is applied in a systematic way to all patients, irrespective of whether or not treatment injection patterns would be tailored and the injection administered differently in clinical practice. Additionally, the relevance of clinical endpoints, which may differ between the trials, must be evaluated and considered within the context of payment for service and real-world treatment expectations.

For each of the discussed studies, the 5-point injection technique for glabellar lines was used, and patients who were enrolled had moderate or severe glabellar lines on the individual manufacturer’s proprietary, validated, 4-point scales, with all studies having patients with moderate to severe lines. Primary endpoints, doses tested, and other study features are listed in Table 1. For each of these studies, response was measured somewhat differently, making comparison difficult.

**Table 1.** Overview of High-Dose BoNTA Studies

Product	Placebo controlled (Y/N)	Maximum dose (X registration dose)	Duration	Volume per injection	Dose studied	Primary endpoint
Abobotulinum (N = ~400)	Yes	2.5	36 weeks (~252 days)	0.05 mL	50U (US label), 75U, 100U, and 125U	Composite response at 30 days (investigator AND patient both report a score of ≤1 in glabellar line severity AND ≥2-grade improvement from baseline)
Incobotulinum (N = 151)	No	3.75	180 days (~28 weeks)	0.05 mL	20U (US label), 50U, and 75U	Return to baseline severity
Onabotulinum (N = 225)	Yes	4.0	48 weeks (336 days)	0.05 mL	20U (US label), 40U, 60U, and 80U	Proportion with ≥1-grade improvement from baseline at week 24 (investigator)

BoNTA, botulinum neurotoxin type A.

In addition to the pitfalls inherent in comparing the results of independent clinical trials, comparison is hindered by the lack of standardized BoNTA units of activity. While many biological products are subject to international standards for measuring activity established by the World Health Organization, no standardized international unit (IU) exists for BoNTAs.^17^ Rather, units of activity for individual BoNTA products are derived from proprietary median lethal dose (LD_50_) assays performed by each product manufacturer.^10^ Thus, true comparisons of products cannot be made using the units defined by each individual manufacturer, and it is impossible to determine if any apparent advantage of one product over another is a result of a higher effective dose, favorable product kinetics, or a mixture of both.

Clinical endpoints are another important consideration for interpreting the effect of high-dose BoNTA. In each of the studies discussed, primary endpoints were either a 1- or 2-point improvement (the defined response rate) on a 4-point scale or time to return to baseline severity. Though the 4-point scales used in these studies are validated, they are not identical, which presents an obstacle to comparison of study results. Furthermore, while investigators are trained extensively on how to use scales (what to do if a patient seems to be “in between” levels or how to apply the scales to patients whose dynamic line patterns are not identical to those shown in the picture, etc.), patients are generally not trained to use the scales, which may cloud interpretation of outcomes reliant upon patient use of these scales.

Realistically, outside of the context of a clinical study, a 1-point improvement for a patient with severe glabellar lines is unlikely to be satisfactory. Rather, in clinical practice, most of the patients are seeking elimination of frown lines from treatment, rather than incremental improvement. For patients with a 2-point response, whether or not the response is satisfactory to the patient may be dependent upon initial baseline severity. The endpoint of return to baseline is also somewhat problematic in that it is exceedingly rare for a patient to wait for dynamic lines to return to baseline before returning for treatment, raising the question of how higher doses of neuromodulators impact time to retreatment, and whether this is a more telling measure of duration. Alternatively, the *loss* of a severity of “none” or “mild” may be used as a surrogate endpoint for retreatment. Endpoints detailing patient satisfaction are most helpful for better understanding the personal, functional outcome of treatment for patients.

### What Is a “High’ Dose?

The concept of “high-dose” BoNTA is one that deserves some discussion. In clinical practice, it is common to adjust dose to suit patient needs. In contrast, the doses used in pivotal trials are generally selected to maximize the chances of regulatory approval and commercial success, rather than to establish a permanent clinical standard. While the approved doses of BoNTA products are indeed efficacious, this does not mean that they are optimal.

The idea of dose modification and optimization is not new. For example, published studies support that male patients and patients with larger muscle mass require a larger BoNTA dose (one panelist noted that she treats the glabellar complex of most female patients with 30U total of ONA, whereas her male patients generally require 80U).^18,19^ Ultrasonographic studies of adult dynamic facial muscles (longitudinal and transverse diameters) show that sex-based differences in the size of mimic musculature are present for the mentalis, depressor anguli oris, and depressor labii inferioris, each of which is significantly smaller in women, as well as for the zygomaticus and frontalis muscles, which are larger in women. In contrast, muscle size is not significantly affected by age and body height,^20^ while body weight is associated, more so in men than in women, with facial muscle size. Though muscle size may not significantly change with age, the pattern of activity can. For example, in the frontalis, shifting patterns generally emerge beginning in the mid-40s. Thus, dosing and injection strategy, even for an individual patient, can change over time.

Within real-world practice, interpatient variability gives rise to a range of responses that are generally not addressed by clinical studies. When considering measures of response duration, it is important not to overlook the fact that durations are reported as means or medians, with many patients experiencing a duration of effect that falls below or above these values. The spectrum of patients encountered in clinical practice will undoubtedly have a range of needs with regard to dose. Importantly, in many BoNTA studies, patients must be treatment-naïve or have not received BoNTA treatment for at least 9 months. Thus, clinical study outcomes are not always reflective of the majority of real-world patients, who often have a long history of treatment. Anecdotally, treatment duration in these long-term patients is longer, most often between 6 and 9 months, with some patients returning for treatment as far out as one year.

Post-approval dose-ranging studies are a critical part of defining the optimal dose and for informing clinician expectations of product behavior, especially for applications such as the treatment of dynamic lines where treatment must be tailored to individual patient anatomy and aesthetic needs; however, individual patient anatomy and needs around treatment duration must be considered.

### Overview of Clinical Study Data

With the above caveats in mind, the authors assessed clinical study outcomes from 3 separate studies on increased dosing for INCO, ONA, and ABO.^13-16^ Primary differences in the studies are presented in **Table 1**.

For ABO, a statistically significant investigator-assessed responder rate (achievement of none or mild) vs placebo was observed through week 28 in the on-label, 50U group (none or mild), and through week 36 in the higher doses of 75, 100, and 125U groups (*P =*< 0.05 vs placebo).^14^ Median time to return to baseline on both investigator and patient 4-point scales was 225 days for 50U, 240 days for 75U, 251 days for 100U, and 256 days for 125U. Based upon the Glabellar Lines Severity Scores, a large majority of patients were satisfied or very satisfied with results at week 24 (82% [50U], 88% [75U], 88% [100U], and 89% [125U]) and at week 36 (67% [50U], 77% [75U], 73% [100U], and 80% [125U]). Importantly, ≥89% of patients reported natural-looking results at all doses and time points up to week 36 (the end of the study). Here, the finding that nearly all responding patients at a given time point perceived the outcome as natural looking is an important finding. Similar affirmation of natural results was evident in the high-dose ONA data, which provides some reassurance to physicians that in patients for whom duration is important, increased dose delivered in small volumes as in these studies is unlikely to lead to a “frozen” look, a common apprehension for patients.

For ONA, intergroup differences for investigator-reported response were statistically significant (*P* =< 0.05) vs the ONA on-label 20U dose, favoring the higher 40U doses of ONA at weeks 1, 16, 20, 24, and 28; ONA 60U at weeks 1, 16, and 20; and ONA 80U at weeks 16, 20, 24, and 28.^13^ At 32 weeks and beyond, differences were not significant between the on-label 20U dose and the higher doses tested. For patient-reported response rates (≥1-grade improvement from baseline), differences compared with ONA 20U were statistically significant at weeks 28 for 40 and 80U. Differences in patient facial line satisfaction survey scores vs ONA 20U were significant at week 24 for 40, 60, and 80U on the questions, “How satisfied are you with how long your treatment results lasted?” and “How satisfied are you that your treatment gave you a natural look?” However, the rate of satisfaction was highest for 40U. Return to baseline was significantly longer for all of the higher doses tested, but the range was narrow, from 19.7 weeks for 20U to 24.1 weeks for 40U, 24.1 weeks for 60U, and 24.0 for 80U. Together, these data reveal that the benefit of increased ONA dose largely plateaus at 40U and that patient satisfaction begins to decrease for higher doses.

For INCO, the median duration of effect (time to return to baseline) was 185 days for 50U, 210 days for 75U, and 177 days for 20U.^16^ These results are similar to those for ONA and ABO, supporting the assertion that increased dose leads to incremental improvements in durability, irrespective of the endpoint measure used. Based on these data, the question is not so much which toxin lasts longest, but whether this incremental change in duration (given that it also appears to give rise to natural-looking results is worth the number of units needed. The answer is likely “it depends on the patient and their priorities,” and these data allow clinicians to have evidence-based discussions with their patients on the likely increase in treatment durability.

### BoNTA Concentration and Injection Volume

An important pattern that emerges from each of the presented high-dose clinical studies is that a more highly concentrated injection of the approved toxin dose appears to have higher efficacy than the same dose administered using larger volume of diluent, as was the case in pivotal trials (eg, 20U in 0.25 mL vs 20U in 0.5 mL). Small studies on the impact of product concentration on treatment effect have yielded different results, with many showing no effect.^21-24^ However, in the high-dose studies, a higher BoNTA concentration has consistently resulted in an increased duration for the on-label registration dose across products. For example, at 150 days (~21.4 weeks) following treatment with 50U ABO in phase III clinical trials, the investigator-assessed ≥1-grade improvement was 13.6%, while for 50U administered at a higher concentration (reconstituted in less diluent) in high-dose studies resulted in a ≥1-grade improvement at 20 weeks of 64% and 53% at 24 weeks.^14,25^ A more concentrated BoNTA injection increases local concentration and effective dose, even for an equal number of units of a given product. This type of microfocused injection likely underpins the low rate of treatment-emergent adverse events (TEAEs) observed in high-dose studies: even with higher dosing, the mocrofocused toxin has a limited field of effect and is most likely to act on the desired muscles with less potential for diffusion and spread. In clinical practice, one limiting factor for microfocused treatment is the need for a fine needle to deliver the BoNTA and a syringe that prevents loss of product. A diabetic syringe may be used to effectively deliver the product in a targeted way but is not ideal to puncture the vial’s cap.

### Safety

Across all high-dose studies, TEAEs were not significantly higher in higher-dose groups. Of note, the duration of ptosis in higher-dose groups was not markedly different from that which occurred in the approved-dose groups. This may be due to the smaller volumes used for treatment and the limited field of effect. The lack of an increased safety signal for higher doses means that for a clinical practice with experienced injectors, patient preference for longer duration of therapy does not require additional education on increased risk of short-term side effects, simplifying discussions and informed decision making.

In addition to these short-term side effects, it is important to consider the potential for an additive impact of high-dose BoNTA on the likelihood a patient will develop neutralizing antibodies (NAbs). Within BoNTA preparations, the toxin itself or the presence of denatured toxin, impurities, or accessory proteins may elicit an immune response.^10^ Though the measurement of NAbs is complex and clinical nonresponse is poorly correlated with the presence of antibodies,^26,27^ the importance of BoNTA in the treatment of serious medical conditions (eg, poststroke spasticity, migraine,^28^ and overactive bladder, among others) means that it is important to preserve BoNTA as a therapeutic option for patients, should they need it. Importantly, more patients are receiving treatments at a younger age and may get these treatments for the next 6 or 7 decades of their lives, a duration far exceeding the periods of time for which data are available. The incidence of NAbs in higher-dose medical indications is thought to increase with dose and shorter intervals^26,29-31^ and may be greater in the presence of BoNTA accessory proteins.^32^ However, the relative risk for doses within the range used for aesthetic indications is undefined, and it is unclear if increasing the number of units for a comparatively low dose indication like glabellar lines, even up to 4 times the registration dose, will have an impact. The proportion of patients who develop NAbs is thought to be between 0% and 1% for aesthetic indications; however, long-term prospective studies are lacking. However, a large meta-analysis of 16 clinical studies with more than 3000 patients found that across aesthetic *and* medical indications, 0.49% (n = 11) of patients seroconverted, with 3 patients having clinical nonresponse at some point following conversion.^28^ Extending the interval between treatments as expected with the longer duration of treatment achievable with higher dosing may prove beneficial for reducing NAb formation. However, whether a higher dose given over less frequent injections is less immunogenic than lower doses given over a greater number of treatment visits is unknown, making it difficult to assess risk of these alternative approaches.

#### Best Practices and Economics

Characterization of optimal doses as “high” is problematic for both physicians and manufacturers. Practice economics are structured around the initial approved dose, and the increased overhead of the high-dose procedure raises an issue that providers will have to navigate. Practices will face the question of whether to pass the expense on (in part or in full) to the patient or to have the cost absorbed by the practice. Across studies, while the number of units is doubled, tripled, or further increased, the duration of effect increases only incrementally. This economic disconnect is one that will be important to remedy before widespread adoption of increased dosing makes economic sense for most of the patients and/or practices.

Though the evidence supports the use of higher-dose toxins, for many patients, the incremental increase in duration may not be sufficient to justify doubling the expense. For treatment-naïve patients, there may be a role for recommending the on-label dose in order to help the patient understand the nature of results that can be expected from treatment.

One limitation of this roundtable is that at the time of this writing, new data on high-dose regimens are continuing to be released and presented at conferences in 5-to 10-minute talks or as posters. As data emerge and are published, and additional analyses are made available, more granular comparisons can be made. However, even at that time, the considerations outlined in this manuscript will be important for evaluating data and applying it to personal clinical practices. In the future, more formal economic analysis can be carried out as well as safety assessments for higher-dose treatments.

### The Future of High-Dose Toxin

The future of BoNTA in aesthetics undoubtedly includes innovation. Novel technologies may be used to change the kinetics of toxin activity and increase duration through entirely different mechanisms unrelated to dose such as extended release. In addition, understanding the drivers of interpatient variability will be an important next step for customizing treatment for individual patients as well as defining the impact of prior treatment on expected results. Finally, the introduction of botulinum toxin E,^33^ an effective toxin with a rapid onset of <24 hours and a short duration of 2 to 4 weeks, raises the question of whether patients can be segmented based on preference for immediate onset and short duration vs a delayed onset (3 days) with a longer duration up through 6 months. The opportunity for providers to be able to offer a range of products and outcomes may help to attract more patients and best meet their desires.

## CONCLUSIONS

Refinement of dose and the concentration of that dose are an important part of helping individual patients achieve their own unique aesthetic goals. Whether the aim is subtotal correction, prevention, or maximum duration, clinical data will continue to help develop a picture of how BoNTA dosing can be modified to optimize patient satisfaction.
